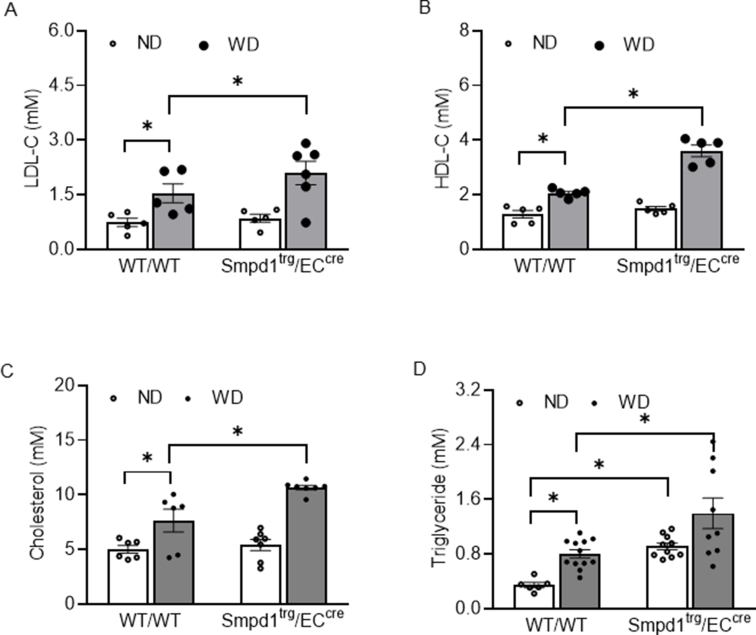# Endothelial Acid Sphingomyelinase Promotes NLRP3 Inflammasome and Neointima Formation During Hypercholesterolemia

**DOI:** 10.1016/j.jlr.2022.100298

**Published:** 2022-10-15

**Authors:** Xinxu Yuan, Owais M. Bhat, Yao Zou, Xiang Li, Yang Zhang, Pin-Lan Li

**Affiliations:** 1 Department of Pharmacology and Toxicology, School of Medicine, Virginia Commonwealth University, Richmond, VA, USA; 2 Department of Pharmacological and Pharmaceutical Sciences, College of Pharmacy, University of Houston, Houston, TX, USA

**Keywords:** carotid artery, endothelial cells, lysosomal ceramide, redox signaling, amitriptyline, NADPH, atherogenesis, 7-ketocholesterol, IL-1β, caspase-1, Ami, amitriptyline, ASC, the adaptor molecule apoptosis-associated speck-like protein containing a CARD, ASM, acid sphingomyelinase, CTXB, cholera toxin B, DHE, dihydroethidium, EC, endothelial cell, FLICA, fluorescent labeled inhibitor of caspase, IHC, immunohistochemistry, IL, interleukin, 7-Ket, 7-ketocholesterol, MR, membrane raft, ND, normal diet, NLRP3, NOD-like receptor pyrin domain 3, NOX, NADPH oxidase, PLCA, partial ligated carotid artery, ROS, reactive oxygen species, α-SMA, α-smooth muscle actin, TXNIP, thioredoxin-interacting protein, vWF, von Willebrand factor, WD, Western diet

## Abstract

The NOD-like receptor pyrin domain 3 (NLRP3) inflammasome is activated during atherogenesis, but how this occurs is unclear. Here, we explored the mechanisms activating and regulating NLRP3 inflammasomes via the acid sphingomyelinase (ASM)-ceramide signaling pathway. As a neointima formation model, partial left carotid ligations were performed on endothelial cell (EC)-specific ASM transgene mice (*Smpd1*^trg^/EC^cre^) and their control littermates (*Smpd1*^trg^/WT and WT/WT) fed on the Western diet (WD). We found neointima formation remarkably increased in *Smpd1*^trg^/EC^cre^ mice over their control littermates. Next, we observed enhanced colocalization of NLRP3 versus adaptor protein ASC (the adaptor molecule apoptosis-associated speck-like protein containing a CARD) or caspase-1 in the carotid ECs of WD-treated *Smpd1*^trg^/EC^cre^ mice but not in their control littermates. In addition, we used membrane raft (MR) marker flotillin-1 and found more aggregation of ASM and ceramide in the intima of *Smpd1*^trg^/EC^cre^ mice than their control littermates. Moreover, we demonstrated by in situ dihydroethidium staining, carotid intimal superoxide levels were much higher in WD-treated *Smpd1*^trg^/EC^cre^ mice than in their control littermates. Using ECs from *Smpd1*^trg^/EC^cre^ and WT/WT mice, we showed ASM overexpression markedly enhanced 7-ketocholesterol (7-Ket)-induced increases in NLRP3 inflammasome formation, accompanied by enhanced caspase-1 activity and elevated interleukin-1β levels. These 7-Ket-induced increases were significantly attenuated by ASM inhibitor amitriptyline. Furthermore, we determined that increased MR clustering with NADPH oxidase subunits to produce superoxide contributes to 7-Ket-induced NLRP3 inflammasome activation via a thioredoxin-interacting protein-mediated controlling mechanism. We conclude that ceramide from ASM plays a critical role in NLRP3 inflammasome activation during hypercholesterolemia via MR redox signaling platforms to produce superoxide, which leads to TXNIP dissociation.

It has been demonstrated that activation of NOD-like receptor pyrin domain 3 (NLRP3) inflammasome is importantly involved in atherogenesis (1). During NLRP3 inflammasome activation, NLRP3 protein as a sensor protein recognizes endogenous and exogenous danger signals and recruits the accumulation of the adaptor protein ASC (the adaptor molecule apoptosis-associated speck-like protein containing a CARD) and the effector protein procaspase-1 to form a proteolytic complex. In such a complex, procaspase-1 is activated to form active caspase-1 leading to the production of mature interleukin (IL)-1β and IL-18 by cleavage of their precursors (2). Recent studies have indicated that endothelial dysfunction and consequent vascular injury and inflammation are associated with the formation and activation of NLRP3 inflammasome (3, 4). In this regard, endothelial NLRP3 inflammasome was found to be activated upon different proatherogenic stimuli such as cholesterol crystals (5), ATP (6), uric acid (7), hyperhomocysteinemia (8), and damage-associated molecular patterns (9) via different pathways. However, the precise mechanisms by which NLRP3 inflammasomes are activated and regulated remain poorly understood.

In general, NLRP3 inflammasome activation has been proposed to relate to increased ionic potassium flux, enhanced production of reactive oxygen species (ROS), and lysosomal rupture (2, 10). Our recent studies have demonstrated the crucial role of ROS-dependent NLRP3 inflammasomes in the regulation of functions of glomeruli or arterial endothelial cells (ECs) and the development of atherosclerosis during hyperlipidemia (3, 11) and the progression of glomerular sclerosis during hyperhomocysteinemia (12, 13, 14). Many endothelial injurious factors including FasL, TNF-α, oxidized LDL, visfatin, and endostatin were found to induce the formation of membrane raft (MR) clusters, in which NADPH oxidase (NOX) subunits such as gp91 and p47 are aggregated to amplify NOX activity in arterial ECs (15). These membrane MR-NOX clusters or complexes that possess a redox signaling function have been referred to as MR redox signaling platforms (16, 17, 18). This led us to hypothesize that the MR redox signaling platforms may play a critical role in endothelial NLPR3 inflammasomes, which have been tested in the present study.

Acid sphingomyelinase (ASM, a lysosomal hydrolase encoded by the *Smpd1* gene) has been shown to importantly participate in the aggregation of NOX subunits in ceramide-enriched MR microdomains promoting the formation of MR redox signaling platform in ECs (15, 19, 20). Upon stimulation, lysosome traffick and fuses to the MR area via a SNARE-centered exocytic machinery (21, 22), where ASM hydrolyzes sphingomyelin into ceramide serving to reorganize and cluster MR-associated signaling molecules (e.g., NOX subunits) in ceramide-enriched MR microdomains (23). Our recent study linked the ASM-MR redox signaling with endothelial NLRP3 inflammasome activation, which was demonstrated by the fact that deficiency of the *Smpd1* gene inhibited the activation of endothelial NLRP3 inflammasomes and neointimal lesion formation in a mouse model of Niemann-Pick disease using *Smpd1* gene global knockout mice (24). The role of ASM-MR redox signaling in NLRP3 inflammasome activation was confirmed in cultured ECs (24). However, the usage of the global gene knockout mouse model could not elucidate whether the ASM-MR redox signaling is derived from an endothelial source that contributes to endothelial NLRP3 inflammasome activation and neointimal lesion formation in vivo. In this study, we generated EC-specific *Smpd1* transgenic mice (*Smpd1*^trg^/EC^cre^) and examined whether the EC-specific overexpression of the *Smpd1* gene could enhance the production of ceramide and the formation of MR redox signaling platforms, promote the activation of endothelial NLRP3 inflammasomes, and thereby result in endothelial dysfunction and atherogenesis. Moreover, using the primary cultured ECs isolated from *Smpd1*^trg^/EC^cre^ mice, we examined whether ASM-MR redox signaling is coupled with endothelial NLRP3 inflammasome activation via a redox sensor, thioredoxin-interacting protein (TXNIP).

## Materials and Methods

### Mice

All animal experiments were performed following the National Institutes of Health guidelines for the care and use of laboratory animals. The protocols were approved by the Institutional Animal Care and Use Committee of Virginia Commonwealth University. Eight- to 12-week-old male and female C57BL/6J WT (WT/WT), *Smpd1*^trg^/WT mice, and EC-specific *Smpd1* transgenic mice were used in the current study. EC-specific *Smpd1* transgenic mice were generated by crossbreeding EC-specific *Cre* transgenic (Tie2 [tunica intima endothelial kinase 2]-Cre) mice with *Smpd1*^trg^/WT mice and genotyped in a similar method as we described for other tissue-specific transgenic mice with *Smpd1* gene overexpression in podocytes (25) and smooth muscle cells (26). Mice were maintained in a controlled environment of 20°C and 40–50% humidity, with a 12-h light/dark cycle. Mice were separated into six groups randomly and fed with the WD (0.21% cholesterol, D12079; Research Diet) for 60 days.

### Partial ligated carotid artery

Partial ligated carotid artery (PLCA) surgery was performed as previously reported by others (27, 28, 29, 30, 31). The surgery was performed after 30 days of WD treatment. Briefly, mice were anesthetized with 2% isoflurane inhalation for 5 min and epilated in the neck and then continued being anesthetized through a nose cone. A ventral midline incision was made in the neck and disinfected with 70% ethanol, and then the muscle layers were separated with curved forceps to expose the left carotid artery after blunt dissection. The external carotid, internal carotid, and occipital artery were ligated with a piece of 6.0 silk suture, whereas the superior thyroid artery was left intact, which provided the sole source for blood circulation. The right carotid artery was not ligated and served as an internal control. After closing the incision and disinfection, the mice were kept on a heating pad until they gained consciousness. After 4 weeks of PLCA, mice were sacrificed, and both their carotid arteries were perfused and isolated for frozen sections and paraffin sections. The slides were used for immunohistochemistry (IHC), dual fluorescence staining, and confocal analysis, respectively.

### Morphologic examination and medial thickening analysis

To study the morphological changes, H&E staining of carotid artery sections was used as described previously (32). Briefly, the carotid artery was perfused with cold PBS for 5 min and 4% cold paraformaldehyde for another 5 min. Then the carotid artery was separated and stored into 10% neutral-buffered formalin. After at least 48 h, the tissues were embedded in paraffin and cut into 5 μm serial sections for histopathological evaluation. For H&E staining, the sections were heated for 10 min at 65°C, and deparaffinization was performed twice in 100% xylene for 10 min. The samples were rehydrated with 100, 95, 90, and 75% ethanol to tap water and immersed in hematoxylin and hydrochloride alcohol. Once the nucleus turned blue, the sections were stained with eosin. After that, the sections were rinsed with running tap water and dehydrated with ethanol of 75, 90, 95, and 100%. Finally, dibutyl phthalate polystyrene xylene was used to mount the slides. Intimal-medium thickening of carotid arteries was measured using Image-Pro Plus 6.0 software (Media Cybernetics, Inc, Bethesda, MD).

### Immunofluorescence staining

Cells cultured on the sterilized cover slides or frozen carotid artery sections were rinsed three times for 2 min with PBS and fixed in 4% paraformaldehyde in PBS for 15 min. Then the samples were washed three times with PBS for 10 min and permeabilizated with 0.1% Triton X-100 in PBS by washing for 10 min followed three times for 5 min with PBS. After that, the samples were blocked with 3% bovine serum albumin for 1 h. Primary antibody dilution and catalog information are NLRP3 (1:200 dilution, catalog no.: MAB7578; R&D Systems), ASC (1:200 dilution, catalog no.: SAB4501314; Santa Cruz), caspase-1 (1:200 dilution, catalog no.: SC-392736; Santa Cruz), flotillin-1 (1:200 dilution, catalog no.: 610820; BD Biosciences; catalog no.: ab41927; Abcam), gp91 (1:200 dilution, catalog no.: 611415; BD Biosciences), p47 (1:200 dilution, catalog no.: 610355; BD Biosciences), ASM (1:200 dilution, catalog no.: LS-C334919; LSBio), cholera toxin B (CTXB; 1:2000 dilution, catalog no.: C34775; Invitrogen), ZO-1 (1:200 dilution, catalog no.: 40-2200; Invitrogen), ZO-2 (1:200 dilution, catalog no.: 71-1400; Invitrogen), von Willebrand factor (vWF; 1:300 dilution, catalog no.: ab11713; Abcam), ceramide (1:200 dilution, catalog no.: ALX-804-196; Enzo). Primary antibodies were incubated overnight at 4°C followed by incubation with secondary antibodies for 1 h at room temperature in the darkroom. Finally, the nucleus was stained by 4',6-diamidino-2-phenylindole and mounted with nail polish. Pictures were taken by a confocal laser scanning microscope (Fluoview FV1000; Olympus, Tokyo, Japan). The fluorescence intensity was measured and analyzed with ImageJ software (National Institutes of Health, Bethesda, MD). The colocalization of NLRP3 with ASC and caspase-1 was analyzed by the Image-Pro Plus, version 6.0 software. These summarized colocalization efficiency data were expressed as Pearson correlation coefficient as described previously (4, 33).

### IHC

To perform the IHC staining, the sections in 10% formalin were dehydrated and embedded in paraffin. Immunohistochemical examinations were performed following the manufacturer's protocol for CHEMICON IHC Select HRP/DAB Kit (EMD Millipore, MA). Briefly, after antigen recovery was performed with pH 6.1 citrate buffer, the sections were blocked by using 3% bovine serum albumin for 1 h at room temperature. After that, primary antibodies (IL-1β, 1:200 dilution, catalog no.: P420B; Invitrogen and α-smooth muscle actin [α-SMA], 1:2000 dilution, catalog no.: ab5694, Abcam) were developed overnight at 4°C and subsequently with biotinylated secondary antibodies and a streptavidin peroxidase complex (PK-7800; Vector Laboratories, Burlingame, CA) for 1 h separately. Finally, the sections were counterstained with hematoxylin and dehydrated as well as mounted using dibutyl phthalate polystyrene xylene. The area percentage of the positive staining was examined using Image-Pro Plus 6.0 software.

### In situ analysis of caspase-1 activity

The caspase-1 activity in the carotid arterial endothelium was analyzed in situ by labeling the active caspase-1 proteins with Fluorescent Labeled Inhibitor of Caspases (FLICA™) probes (ImmunoChemistry Technologies, LLC, Bloomington, MN) as described previously (34). The FLICA probes are comprised of three moieties including a caspase-1 recognition sequence tyrosine-valine-alanine-aspartic acid (YVAD) that binds to active caspase-1, a fluoromethyl ketone moiety that results in irreversible binding with the enzyme, and a fluorescent tag carboxyfluorescein reporter. After entering the cells, the FLICA reagent carboxyfluorescein-YVAD-fluoromethyl ketone becomes covalently coupled to the active caspase-1, whereas any unbound FLICA reagent diffuses out of the cell and is washed away. The remaining green fluorescent signal is a direct measure of the active caspase-1 enzyme activity in the cell or tissue samples. To detect caspase-1 activity in the carotid arterial endothelium, frozen artery section slides were first fixed in acetone and incubated overnight at 4°C with sheep anti-vWF (1:200 dilution; Abcam, Waltham, MA). These slides were then costained with fluorescence-conjugated anti-sheep secondary antibody and FLICA reagent (1:10 dilution) for 1.5 h at room temperature, washed, mounted, visualized, and analyzed by confocal microscopy as described previously.

### Cell culture

Isolation of mouse carotid arterial ECs was performed and characterized as previously described (15). ECs were cultured in DMEM (Gibco), supplemented with 10% FBS (Gibco) and 1% penicillin-streptomycin (Gibco) in humidified 100% air and 5% CO_2_ mixture at 37°C. ECs were primed with a low dose of lipopolysaccharide (1 ng/ml) for 3 h before any experiments. For the proatherogenic stimulation, cells were treated with 7-ketocholesterol (7-Ket; 15 μg/ml) and then incubated for 21 h. In the case of inhibitors used, the cells were pretreated with amitriptyline (Ami) (20 μM) for 30 min.

### Western blot analysis

Western blot analysis was performed as we described previously (35). Briefly, whole-cell lysate protein concentrations were measured and resuspended to 2 μg/μl. Cell lysates were run on an SDS-PAGE gel at a voltage of 100 V for 2 h and transferred into a polyvinylidene difluoride membrane at a voltage of 100 V for 1 h. The membrane was blocked with 5% nonfat milk for 1 h, followed by incubation with the following primary antibodies: cleaved caspase-1 (1:500 dilution; Santa Cruz) overnight at 4°C. Then the secondary antibody was labeled with HRP for 1 h at room temperature, and the membrane was washed three times with Tris-buffered saline with Tween-20. The intensity of the bands was quantified using ImageJ 6.0.

### ELISA analysis of IL-1β secretions

The fresh culture medium was collected for IL-1β product measurement with an IL-1β ELISA kit according to the manufacturer's instructions as we described (36). In brief, at least 200 μl of the culture medium was used and incubated for 2 h at room temperature. Then, the samples were incubated with IL-1β conjugate for another 2 h at room temperature. Thorough washes were performed between and after the two incubations. About 100 μl of substrate solution was applied to generate chemiluminescence. Chemiluminescent absorbance was examined using a microplate reader at λ = 450.

### ESR analysis of O_2_•^−^ production

ESR detection of O_2_•^−^ was performed as previously described (37). Briefly, cellular protein concentrations were measured in a lysis buffer and then prepared for analysis by resuspension in a modified Kreb's-Hepes buffer containing deferoxamine (100 μM) and diethyldithiocarbamate (5 μM). NOX-dependent O_2_•^−^ production was examined by the addition of 1 mM NADPH as a substrate in 20 μg protein in the presence or the absence of superoxide dismutase (200 U/ml) to produce O_2_•^−^. Then, 10 mM 1-hydroxy-3-methoxycarbonyl-2,2,5,5-tetramethylpyrrolidine, a superoxide-specific spin-trapping compound, was added to trap O_2_•^−^ before the mixture was loaded into the glass capillaries and immediately measured the O_2_•^−^ production kinetically for 10 min using a Miniscope MS200 ESR spectrometer (Magnettech, Germany). The ESR settings were as follows: biofield, 3,350; field sweep, 60 G; microwave frequency, 9.78 GHz; microwave power, 20 mW; modulation amplitude, 3G; 4,096 points of resolution; receiver gain, 100; and kinetic time, 10 min. Superoxide dismutase-sensitive components of ESR signals were used to calculate changes in O_2_•^−^ production or its level, which were shown as the fold changes of control.

### In situ O_2_•^−^ production in mouse carotid arteries

Dihydroethidium (DHE) is a lipophilic cell-permeable dye that is one of the most widely used fluorogenic probes for the detection of intracellular superoxide (38). In brief, the frozen tissue slides were incubated with 10 mM DHE in phosphate-buffered saline at room temperature for 30 min. Then, the slides were washed, fixed, and subjected to confocal microscopic analysis (Fluoview FV1000; Olympus).

### Trichrome stain in mouse carotid arteries

Trichrome stain (Abcam; catalog no.: ab150686) was used to detect extracellular matrix and fibrosis following the manufacturer’s instructions. Deparaffinize sections and hydrate in distilled water. Preheat Bouin’s fluid in a water bath to 56–64°C in a fume hood or very well-ventilated area. Place slide in preheated Bouin’s fluid for 60 min followed by a 10-min cooling period. Rinse the slide in tap water until the section is completely clear. Rinse once in distilled water. Mix equal parts of Weigert’s (A) and Weigert’s (B) and stain the slide with working Weigert’s iron hematoxylin for 5 min. Rinse slide in running tap water for 2 min. Apply Biebrich Scarlet/Acid Fuchsin solution to slide for 15 min. Rinse the slide in distilled water. Differentiate in phosphomolybdic/phosphotungstic acid solution for 10–15 min or until the collagen is not red. Without rinsing, apply Aniline Blue solution to the slide for 5–10 min. Rinse the slide in distilled water. Apply acetic acid solution (1%) to slide for 3–5 min. Dehydrate very quickly in two changes of 95% alcohol, followed by two changes of absolute alcohol. Clear in xylene or xylene substitute and mount in synthetic resin. The images are quantified using ImageJ 6.0.

### ASM activity assay

ASM activity was measured using the Acid Sphingomyelinase Assay Kit (catalog no.: ab190554; Abcam) following the manufacturer’s instructions. The WT/WT ECs were treated with different concentrations of Ami (Ctrl, 10, 20, 40 μM). Then cell homogenization was used for the activity assay.

### Statistics

Data are presented as means ± SEM. Significant differences between and within multiple groups were examined using ANOVA for repeated measures, followed by Duncan's multiple range test. The statistical analysis was performed by Sigmaplot 12.5 software (Systat Software, San Jose, CA). *P* < 0.05 was considered statistically significant.

## Results

### EC-specific overexpression of the *Smpd1* gene exacerbates atherosclerotic lesions in mouse carotid arteries

**Fig. 1:**
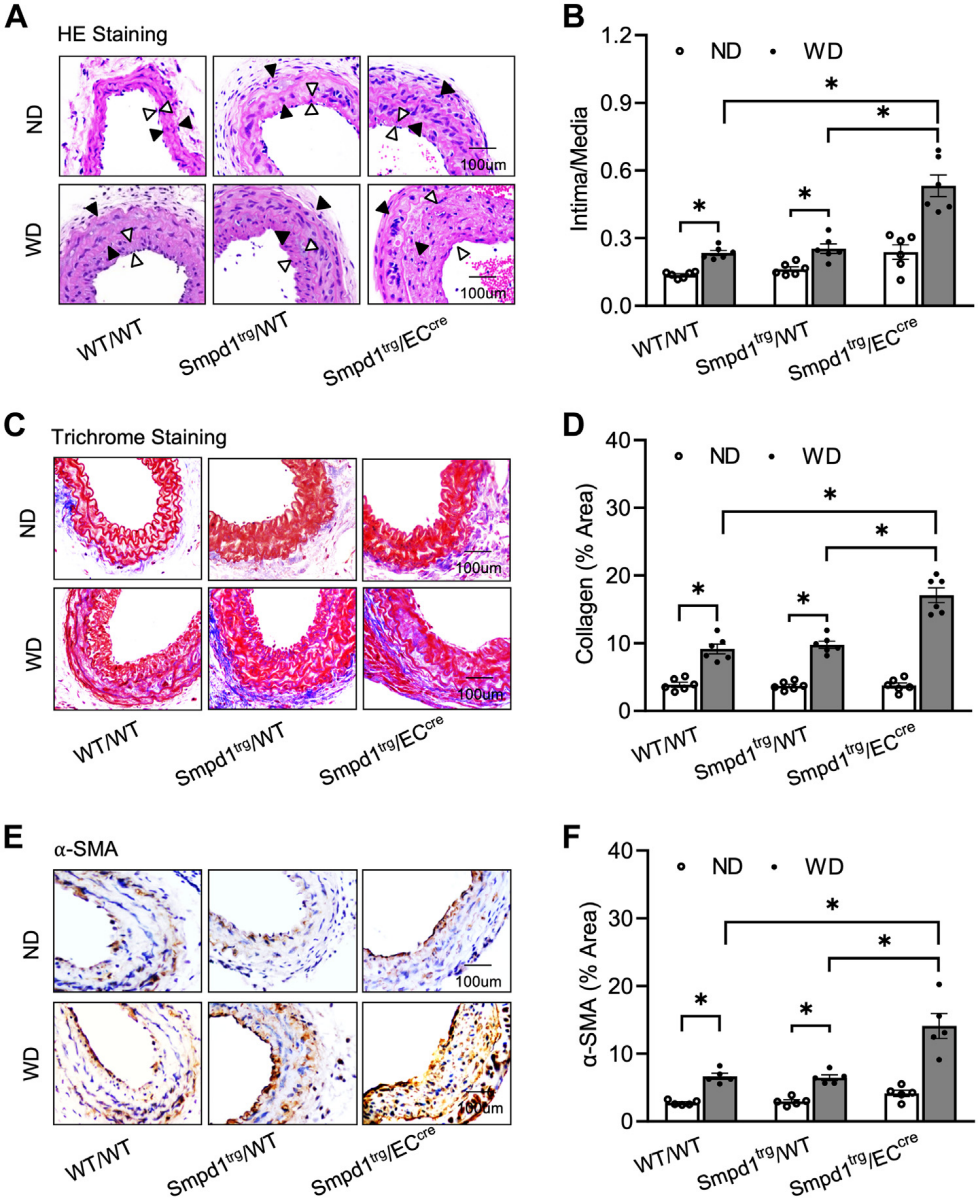
Atherosclerotic lesions in the carotid arteries in EC-specific *Smpd1* transgenic mice during hypercholesterolemia. A: H&E staining showing the neointima and media on the mouse carotid arterial wall. AOI: the media area (black arrowheads) and the intima area (white arrowheads). B: Quantitative analysis of atherosclerotic lesions in PLCA represented by calculation of the ratio between arterial intima and media area. C: Trichrome staining showing the collagen deposition on the mouse carotid arterial wall. D: Quantification of the blue color of Trichrome staining. E: Representative microscopic images of tissue slide with immunohistochemical staining that shows α-SMA expression on the arterial wall. F: Summarized data showing the density of α-SMA stained with anti-α-SMA antibody. Data are expressed as means ± SEM, *n* = 5. ∗*P* < 0.05 is defined as significant. The scale bar represents 100 μm.

To induce neointima formation in carotid arteries, a PLCA model was surgically prepared in mice fed with a Western diet (WD) for 4 weeks as we described previously (3, 24, 39). Two control mice were used in our studies including WT (WT/WT) mice and floxed *Smpd1* transgene (*Smpd1*^trg^/WT) mice. The EC-specific *Smpd1* transgene (*Smpd1*^trg^/EC^cre^) mice were generated by crossing *Smpd1*^trg^/WT mice with EC-specific Tie2-Cre mice as described (40). Tie2 promoter-driven Cre recombinase activity results in overexpression of the *Smpd1* gene in ECs. We first confirmed the animal model by measuring the serum lipids (LDL-C, HDL-C, total cholesterol, and triglyceride) and found that *Smpd1*^trg^/EC^cre^ mice enhanced the lipid levels induced by the WD (supplemental Fig. S3A–D). As shown in Fig. 1A, the neointima formation was not observed in PLCAs of normal diet (ND)-treated WT/WT mice or *Smpd1*^trg^/WT mice. ND-treated *Smpd1*^trg^/EC^cre^ mice exhibited mild neointima thickening, but there is no statistically significant increase in the ratio of intima/media (Fig. 1A, B). In contrast, WD treatment significantly increased the neointima formation and the intima/media ratio in PLCAs of WT/WT and *Smpd1*^trg^/WT mice, which were more significantly enhanced in *Smpd1*^trg^/EC^cre^ mice (Fig. 1A). These data indicate that hypercholesterolemia-induced neointimal lesions were exacerbated when the *Smpd1* gene is overexpressed in ECs.

To further investigate the histological changes in the carotid arteries of all groups, Masson’s trichrome staining and α-SMA expression on the carotid wall by IHC staining was performed. We also found that WD treatment significantly showed active collagen production (Fig. 1C, D) and increased α-SMA expression (Fig. 1E, F) on the carotid media and intima layer of WT/WT and *Smpd1*^trg^/WT mice, which were more significantly enhanced in *Smpd1*^trg^/EC^cre^ mice.

**Figure S1:**
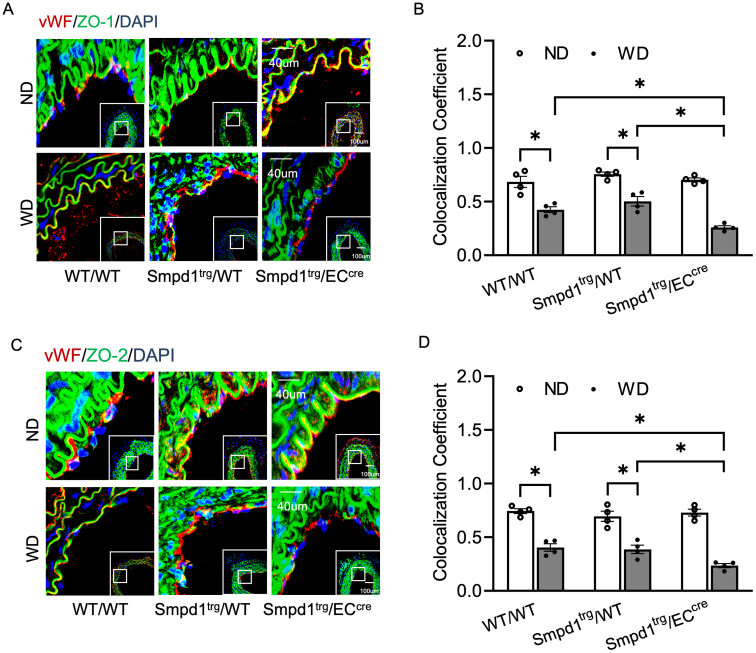


We also studied the effects of ASM overexpression on the endothelial injury. As shown in supplemental Fig. S1A, C, intact endothelium in ND-treated mice expresses similar higher ZO-1 and ZO-2 than in WD-treated mice; however, *Smpd1*^trg^/EC^cre^ mice significantly attenuated WD-induced decrease. supplemental Fig. S1B, D showed clear changes.

### EC-specific overexpression of the *Smpd1* gene enhances endothelial inflammasome activation in the carotid arteries

**Fig. 2:**
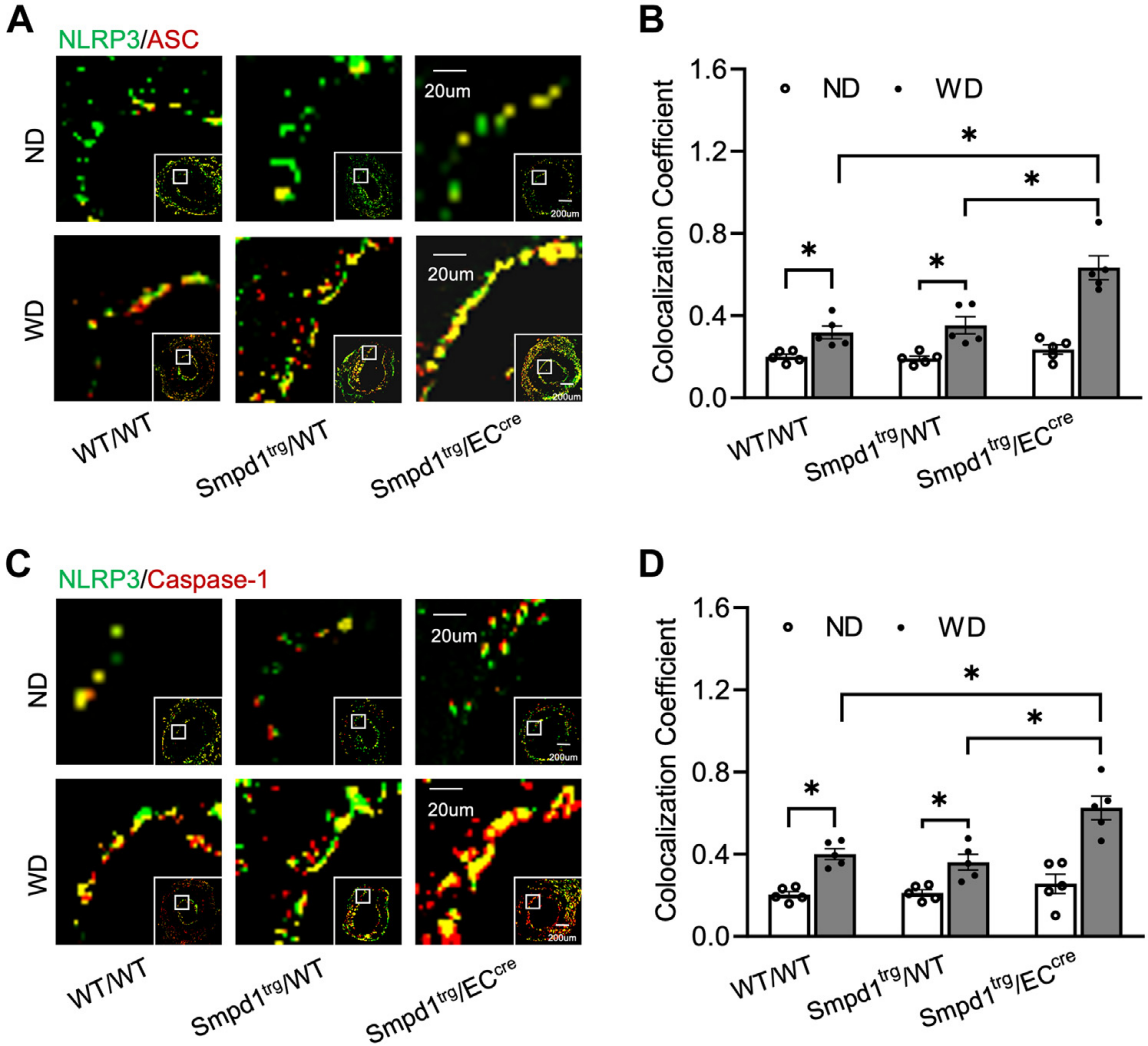
Endothelial NLRP3 inflammasome formation in the carotid arteries of endothelium-specific *Smpd*1 transgenic mice during hypercholesterolemia. A: Representative fluorescent confocal microscope images displaying the yellow dots or patches showing the colocalization of NLRP3 (green) with ASC (red). B: The summarized data showing the colocalization coefficient of NLRP3 with ASC. C: Representative fluorescent confocal microscope images showing the colocalization of NLRP3 (green) with caspase-1 (red). D: The summarized data show the colocalization coefficient of NLRP3 with caspase-1. Data are expressed as means ± SEM, *n* = 5. ∗*P* < 0.05 is defined as significant. The scale bar represents 20 μm or 200 μm.

**Fig. 3:**
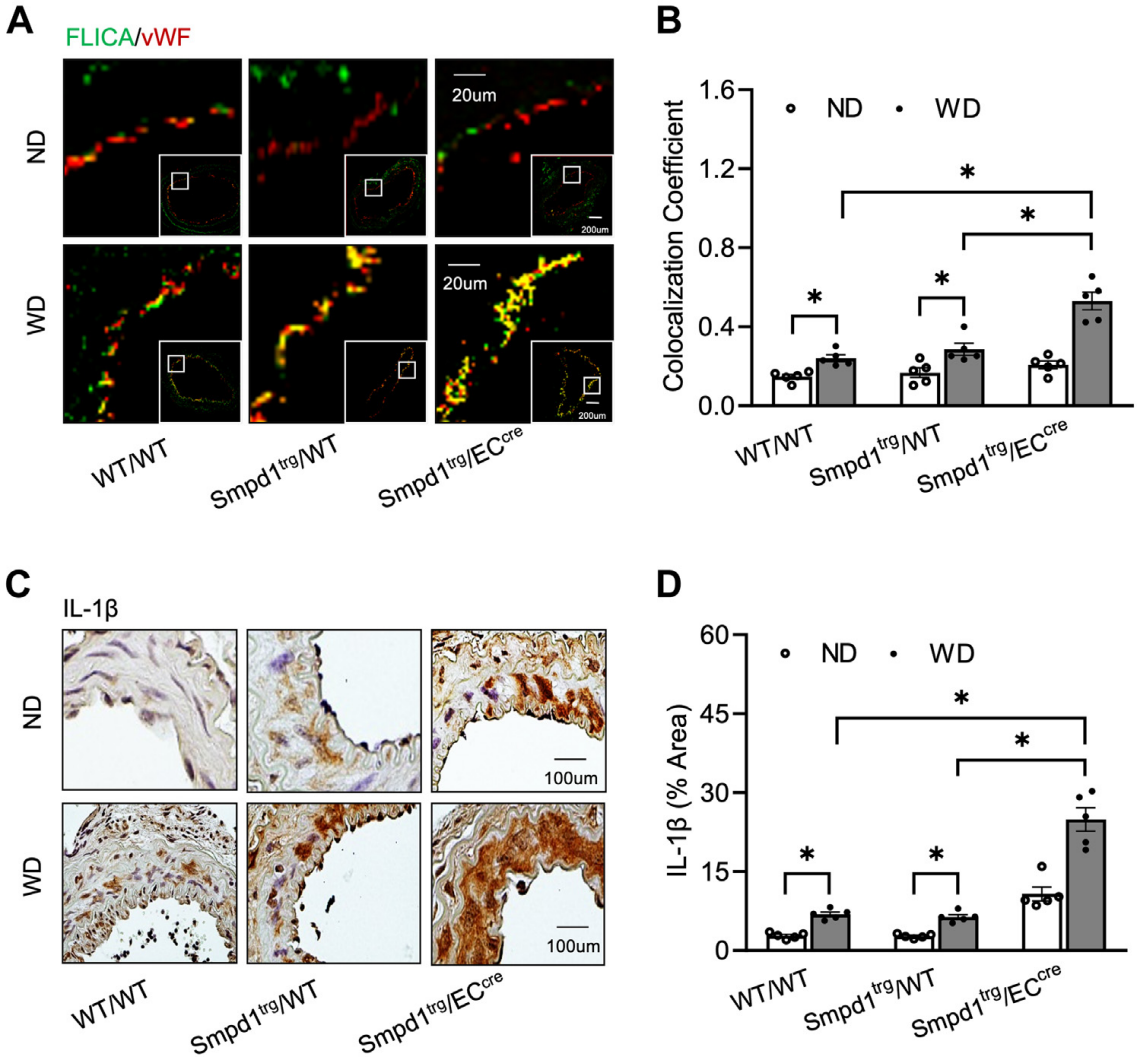
Endothelial NLRP3 inflammasome activation in the carotid arteries of EC-specific *Smpd1* transgenic mice during hypercholesterolemia. A: Representative fluorescent confocal microscope images displaying the yellow dots or patches showing the colocalization of FLICA (green) with vWF (red). B: The summarized data show the colocalization coefficient of FLICA with vWF. C: Representative microscopic images of tissue slide with immunohistochemical staining that shows IL-1β accumulation on the arterial wall. D: Summarized data showing the density of IL-1β stained with selective anti-IL-1β antibody. Data are expressed as means ± SEM, *n* = 5. ∗*P* < 0.05 is defined as significant. The scale bar represents 20 μm or 200 μm for A and 100 μm for C.

The NLRP3 inflammasome formation in PLCAs was analyzed by examining the colocalization of NLRP3 inflammasome subunits using confocal immunofluorescence microscopy. As shown in Figure 2A, C, in WT or *Smpd1*^trg^/WT control mice, WD significantly increased the colocalization of NLRP3 with ASC or caspase-1 compared with ND as shown by yellow spots in the intima of the carotid arteries. Moreover, the WD-induced increase in the colocalization of NLRP3 subunits was enhanced in *Smpd1*^trg^/EC^cre^ mice compared with WT/WT or *Smpd1*^trg^/WT mice. The quantified colocalization coefficient data are summarized in Figure 2B, D. In addition to their formation, the activation of the NLRP3 inflammasome was analyzed by examining the caspase-1 activity using FLICA probes (Fig. 3A, B) and IL-1β expression using IHC staining (Fig. 3C, D). As shown in Figure 3A, B, there was no significant colocalization of FLICA with endothelial marker vWF in all mouse strains with ND treatment suggesting a basal level of NLRP3 inflammasome activity in these mice. In contrast, WD treatment significantly increased the FLICA/vWF colocalization in WT/WT and *Smpd1*^trg^/WT control mice, and such WD-induced colocalization was more significantly increased in *Smpd1*^trg^/EC^cre^ mice. Accompanied by enhanced caspase-1 activity by WD, *Smpd1*^trg^/EC^cre^ mice showed a more significant increase in the levels of IL-1β expression by WD in the carotid intima region compared with that in WT/WT and *Smpd1*^trg^/WT mice (Fig. 3C, D). Together, these data suggest that EC-specific *Smpd1* gene overexpression enhances endothelial NLRP3 inflammasome formation and activation in the carotid arteries of mice during hypercholesterolemia.

### EC-specific overexpression of *Smpd1* gene amplifies ceramide-MR-mediated redox signaling in the carotid artery walls

**Fig. 4:**
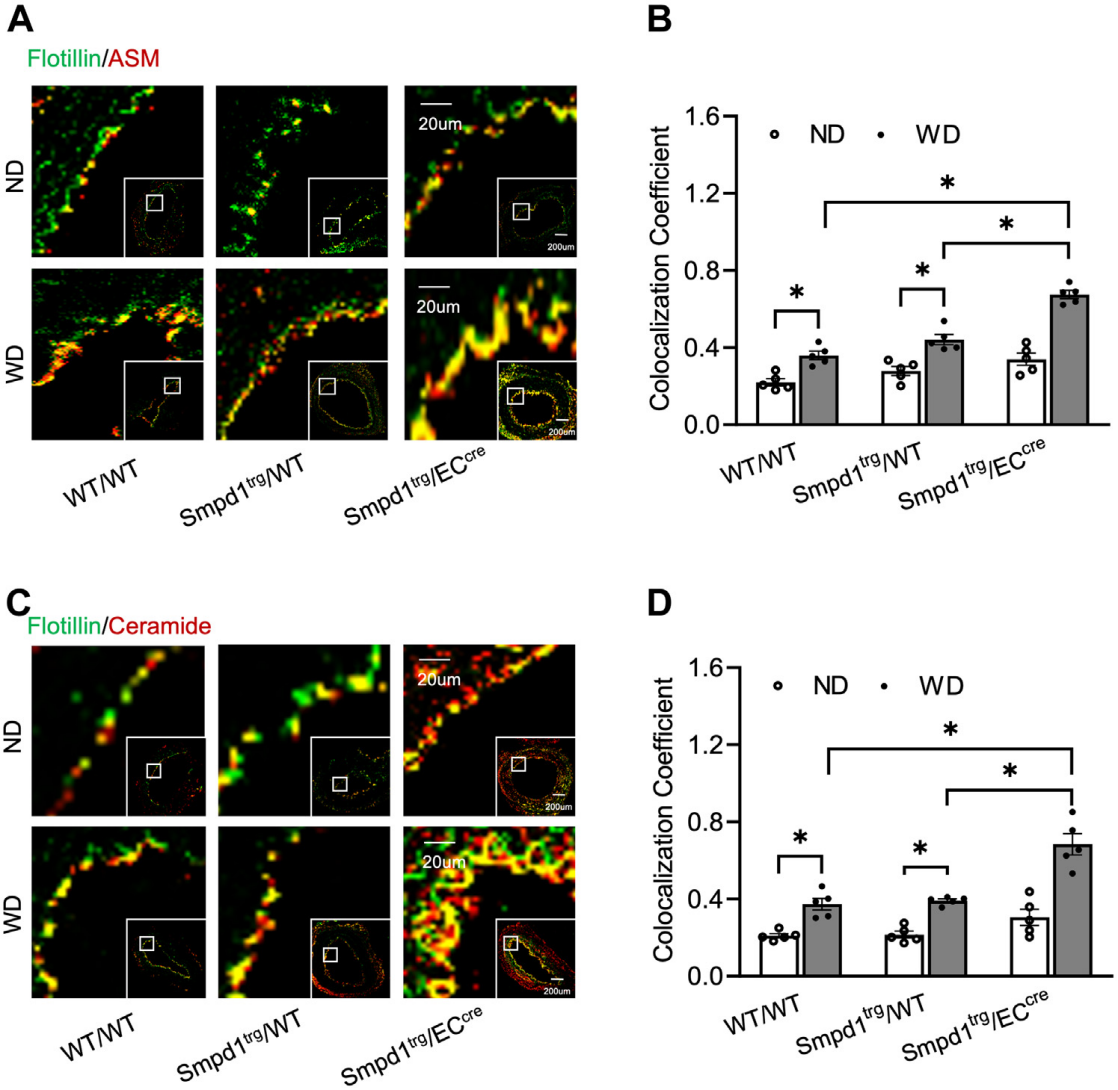
Accumulation of ASM and ceramide in endothelial membrane rafts on the carotid artery wall of EC-specific *Smpd1* transgenic mice during hypercholesterolemia. A: Representative fluorescent confocal microscopic images showing the colocalization of MR marker, flotillin (green) with ASM (red). B: Summarized data showing the colocalization coefficient of flotillin with ASM. C: Representative fluorescent confocal microscopic images showing the colocalization of MR marker, flotillin (green) with ceramide (red). D: Summarized data showing the colocalization coefficient of flotillin with ceramide. Data are expressed as means ± SEM, *n* = 5. ∗*P* < 0.05 as defined is significant. The scale bar represents 20 μm or 200 μm.

Earlier reports from our laboratory have demonstrated that ASM plays an essential role in forming ceramide-enriched MR clusters in ECs, in which NOX subunits are aggregated and activated leading to redox signaling and consequent endothelial NLRP3 inflammasome activation and arterial neointima formation during hypercholesterolemia (18, 24). Here, we observed that WD treatment increased the colocalization of ASM or ceramide within flotillin (MR marker) in the intima region of PLCAs of WT/WT or *Smpd1*^trg^/WT mice compared with ND-treated controls, whereas such increases were further enhanced in *Smpd1*^trg^/EC^cre^ mice (Fig. 4A, C). These changes were quantified as a colocalization coefficient as summarized in Figure 4B, D. It should be noticed that under basal conditions (ND groups), *Smpd1* gene overexpression only caused a statistically insignificant increase in either the ASM protein expression or ceramide in MR clusters in the carotid intima of *Smpd1*^trg^/EC^cre^ mice compared with WT/WT or *Smpd1*^trg^/WT mice (Fig. 4A–D). Thus, our data suggest that EC-specific overexpression of the *Smpd1* gene sensitizes the hypercholesterolemia-induced formation of ceramide-enriched MR clusters in the carotid arterial intima.

**Fig. 5:**
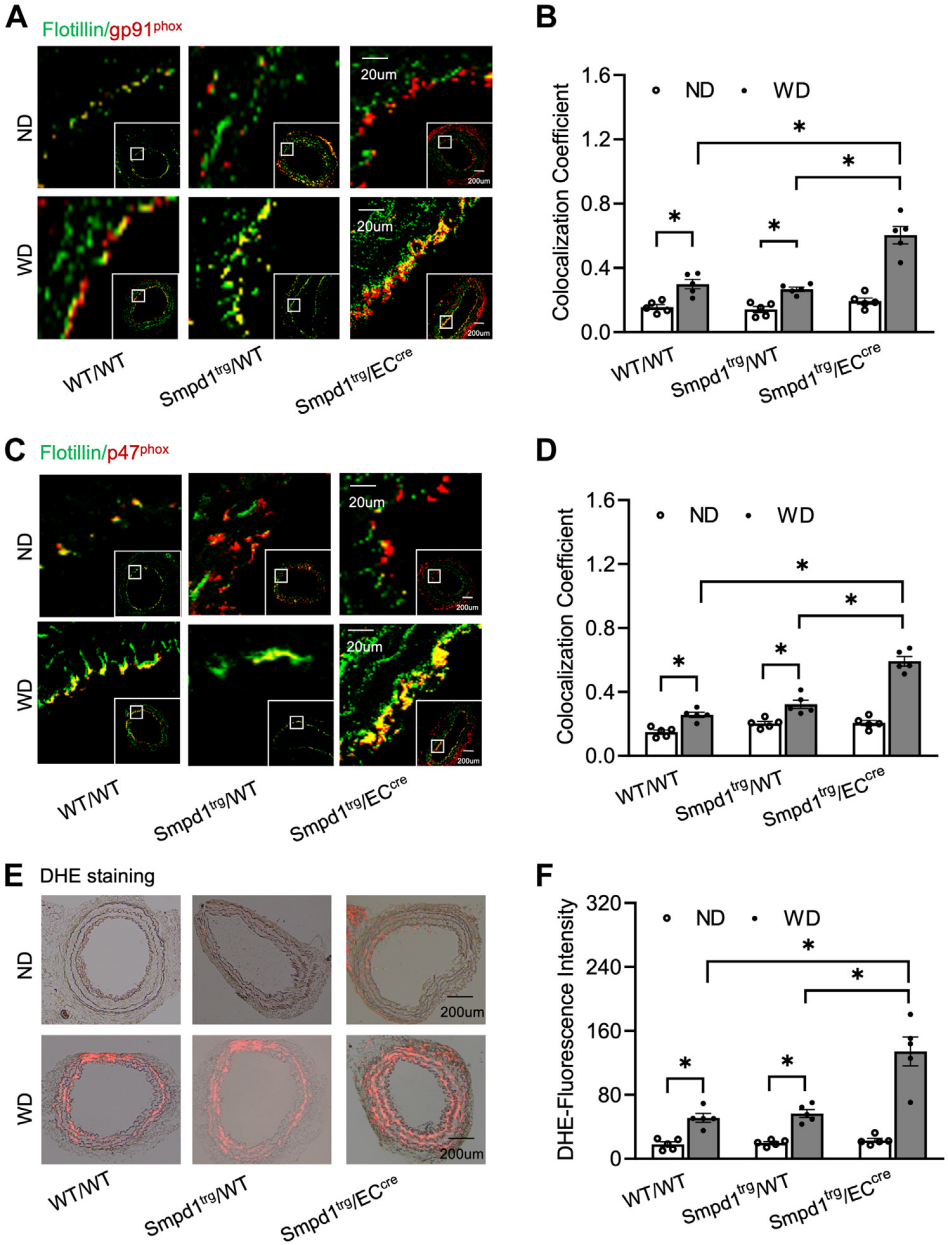
NOX subunit aggregation and superoxide production in the carotid artery wall during hypercholesterolemia. A: Representative fluorescent confocal microscopic images showing the colocalization of MR marker, flotillin (green) with NOX subunit, gp91^phox^ (red). B: Summarized data showing the colocalization coefficient of flotillin with gp91^phox^. C: Representative fluorescent confocal microscopic images showing the colocalization of flotillin (green) with NOX subunit, gp47^phox^ (red). D: Summarized data showing the colocalization coefficient of flotillin with gp47^phox^. E: Representative merged images (DHE red fluorescence merged with transmission light) showing the O_2_•^−^ production in situ in mouse carotid arteries. F: Summarized data showing the DHE fluorescence intensity. Data are expressed as means ± SEM, *n* = 5. ∗*P* < 0.05 is defined as significant. The scale bar represents 20 μm or 200 μm for A and C and 200 μm for E.

Consistent with the increased formation of ceramide-enriched MR clusters by WD in *Smpd1*^trg^/EC^cre^ mice, we also observed that WD induced more significant increases in the aggregation of NOX subunits gp91 or p47 in ceramide-enriched MR clusters in these mice (Fig. 5A–D). Furthermore, O_2_•^−^ production in situ was detected by DHE staining on the carotid arterial wall. As shown in Figure 5E, F, WD caused a more significant increase in DHE-O_2_•^−^ signal in *Smpd1*^trg^/EC^cre^ mice compared with that in WT/WT or *Smpd1*^trg^/WT mice. Together, these data suggest that hypercholesteremia-induced MR-redox signaling is amplified in the carotid arterial wall of mice when the *Smpd1* gene is overexpressed in ECs.

### *Smpd1* gene overexpression enhances NLRP3 inflammasome formation and activation in cultured ECs

**Fig. 6:**
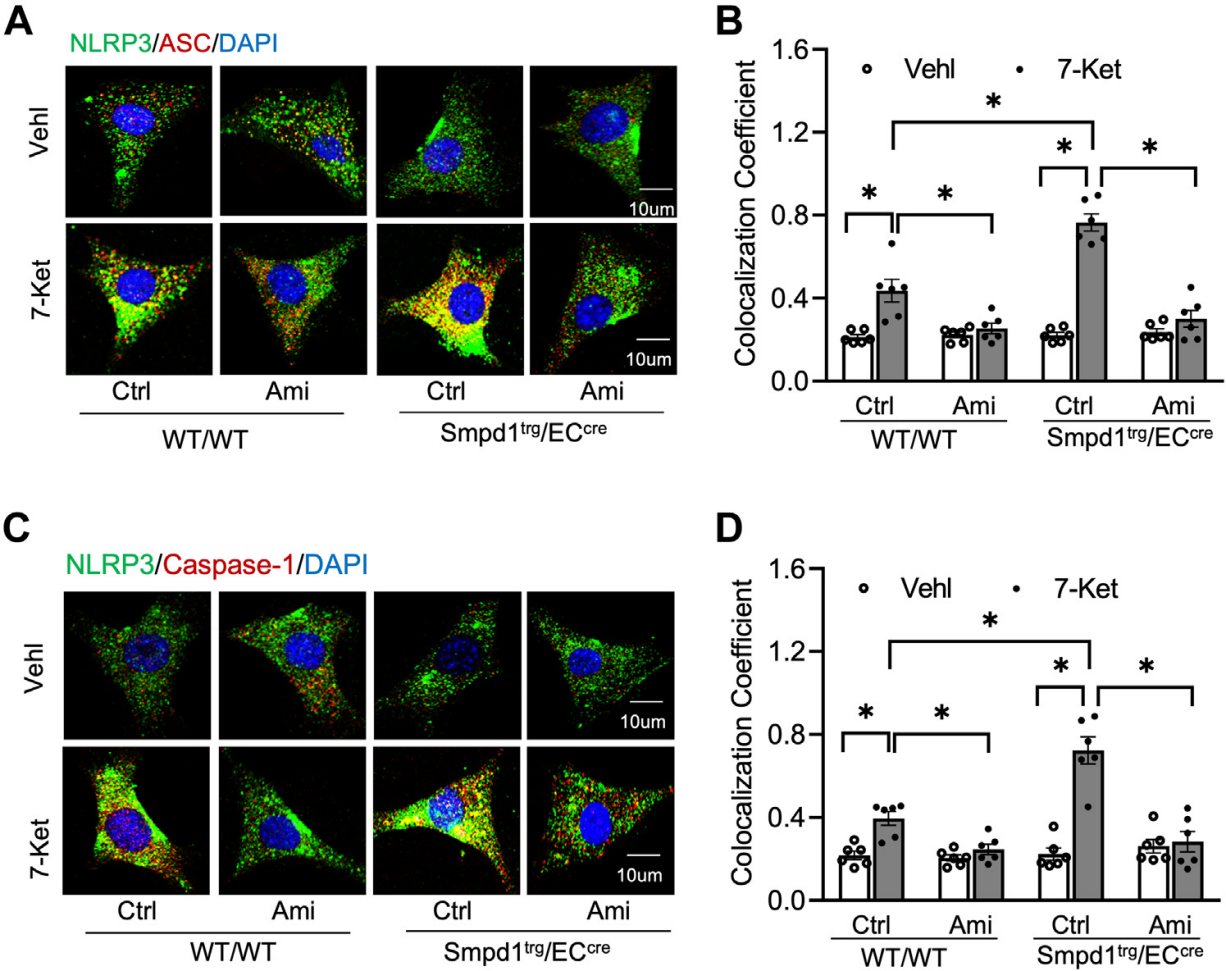
Effects of ASM blockade on NLRP3 inflammasome formation in the primary cultures of ECs from EC-specific *Smpd1* transgenic mice. Primary cultured carotid arterial ECs were treated with 7-Ket (15 μg/ml) for 24 h followed by treatment of ASM inhibitor Ami (20 μmol/l). A: Representative fluorescent confocal microscope images displaying the yellow dots or patches showing the colocalization of NLRP3 (green) with ASC (red). B: The summarized data show the colocalization coefficient of NLRP3 with ASC. C: Representative fluorescent confocal microscope images showing the colocalization of NLRP3 (green) with caspase-1 (red). D: The summarized data show the colocalization coefficient of NLRP3 with caspase-1. Data are expressed as means ± SEM, *n* = 6. ∗*P* < 0.05 is defined as significant. The scale bar represents 10 μm.

**Figure S2:**
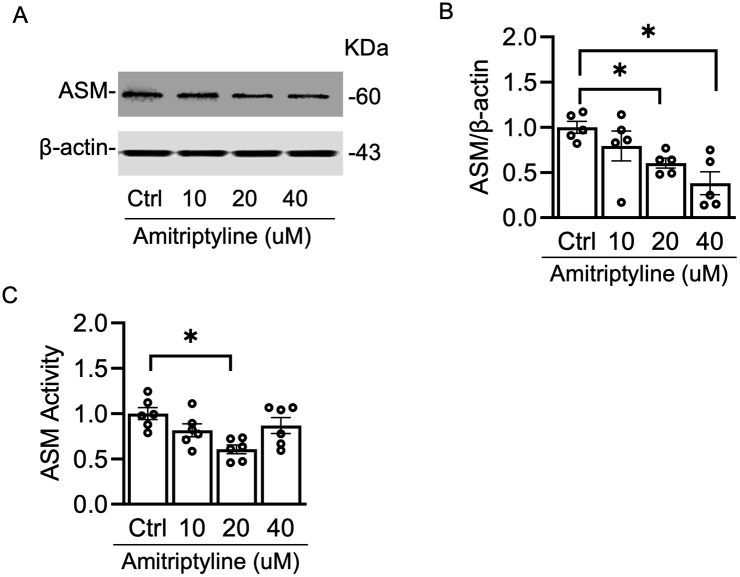


Next, we aimed to confirm the contribution of *Smpd1* gene overexpression to NLRP3 inflammasome activation and formation in primary cultured ECs from carotid arteries of WT/WT and *Smpd1*^trg^/EC^cre^ mice as previously described (41, 42). First, we confirmed that Ami, an ASM inhibitor, dose-dependently decreased ASM protein by Western blot and ASM activity detected by ELISA kit (supplemental Fig. S2A–C). Next, it was found that 7-Ket-induced colocalization of NLRP3 with ASC or caspase-1 was more significantly increased in *Smpd1*^trg^/EC^cre^ ECs compared with that in WT/WT ECs, whereas pretreatment of both ECs with Ami, an ASM inhibitor, abolished such 7-Ket-induced increases (Fig. 6A–D).

**Fig. 7:**
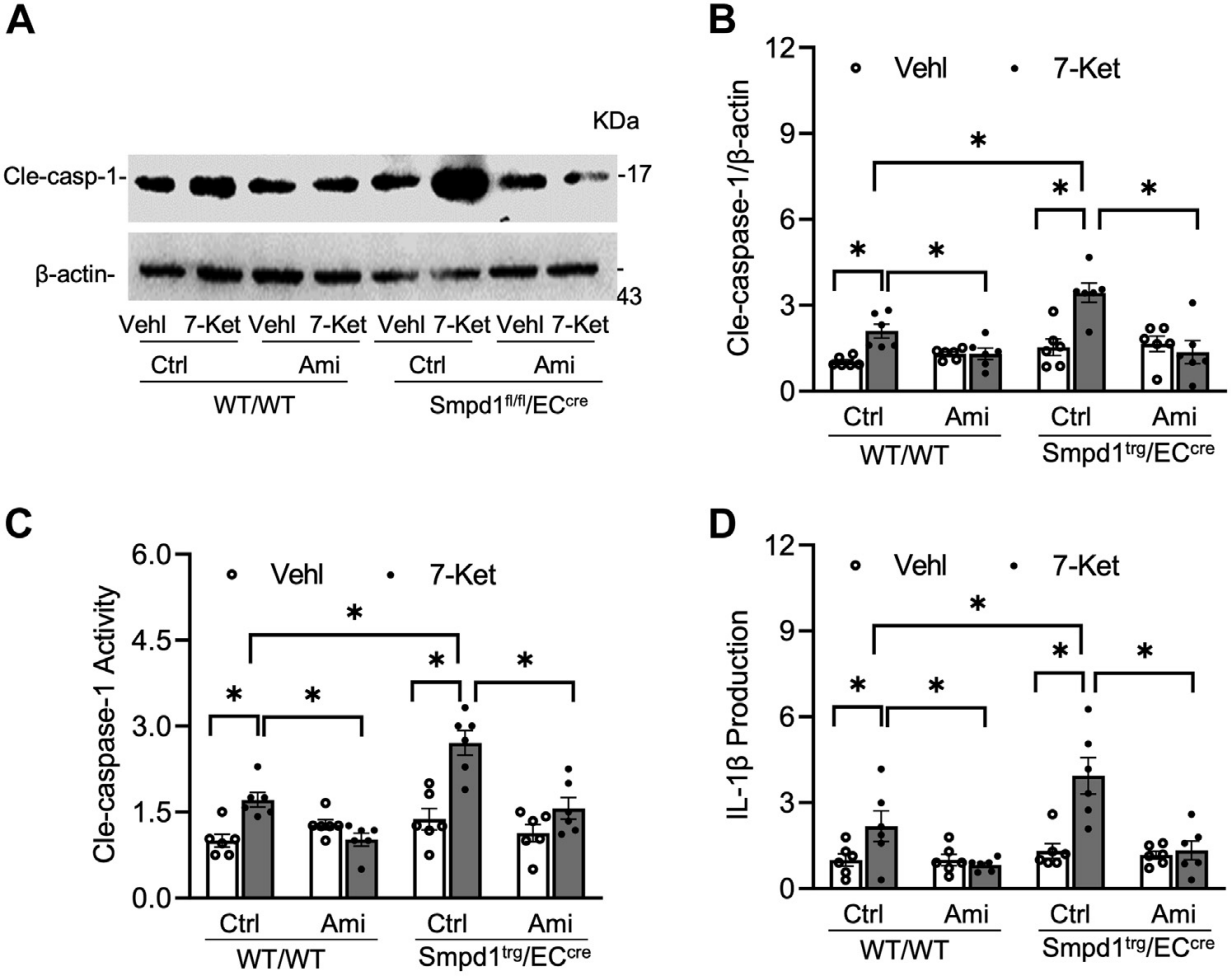
Effects of ASM blockade on NLRP3 inflammasome activation in the primary cultures of ECs from EC-specific *Smpd1* transgenic mice. A: Representative Western blot gel documents showing the expression of cleaved caspase-1 in the primary cultures of carotid ECs from endothelium-specific *Smpd1* transgenic mice. B: Summarized data showing the expression of cleaved caspase-1 in the primary cultures of carotid ECs from endothelium-specific *Smpd1* transgenic mice. C: Summary data showing caspase-1 activity in the primary cultures of carotid ECs from endothelium-specific *Smpd1* transgenic mice. D: Summarized data showing IL-1β production in the primary cultures of carotid ECs from endothelium-specific *Smpd1* transgenic mice. Data are expressed as means ± SEM, *n* = 5. ∗*P* < 0.05 is defined as significant.

The NLRP3 inflammasome activation in cultured ECs was further assessed by Western blot analysis of the caspase-1 cleavage, caspase-1 activity assay, and ELISA analysis of IL-1β production. As shown in Figure 7A, B, 7-Ket remarkably increased cleaved caspase-1 expression in WT/WT ECs, which was further enhanced in *Smpd1*^trg^/EC^cre^ ECs. Such enhancement in caspase-1 cleavage in Smpd1^trg^/EC^cre^ ECs was correlated with increased caspase-1 activity (Fig. 7C) and IL-1β production (Fig. 7D). Moreover, 7-Ket-induced increases in caspase-1 cleavage, caspase-1 activity, and IL-1β production were prevented by Ami in both WT/WT and *Smpd1*^trg^/EC^cre^ ECs (Fig. 7A–D). It should be noticed that under basal conditions (vehicle), *Smpd1*^trg^/EC^cre^ ECs did not have significantly higher levels of NLRP3 inflammasome formation (Fig. 6A–D) and activation (Fig. 7A–D) compared with WT/WT ECs. Therefore, *Smpd1* gene overexpression alone seems to be insufficient to trigger NLRP3 inflammasome formation and activation. However, our data indicate that *Smpd1* gene overexpression in ECs could enhance 7-Ket-induced NLRP3 inflammasome formation and activation in vitro, which is large because of increased ASM activity.

### *Smpd1* gene overexpression enhances the formation and activation of MR-redox signaling platforms in cultured ECs

**Fig. 8:**
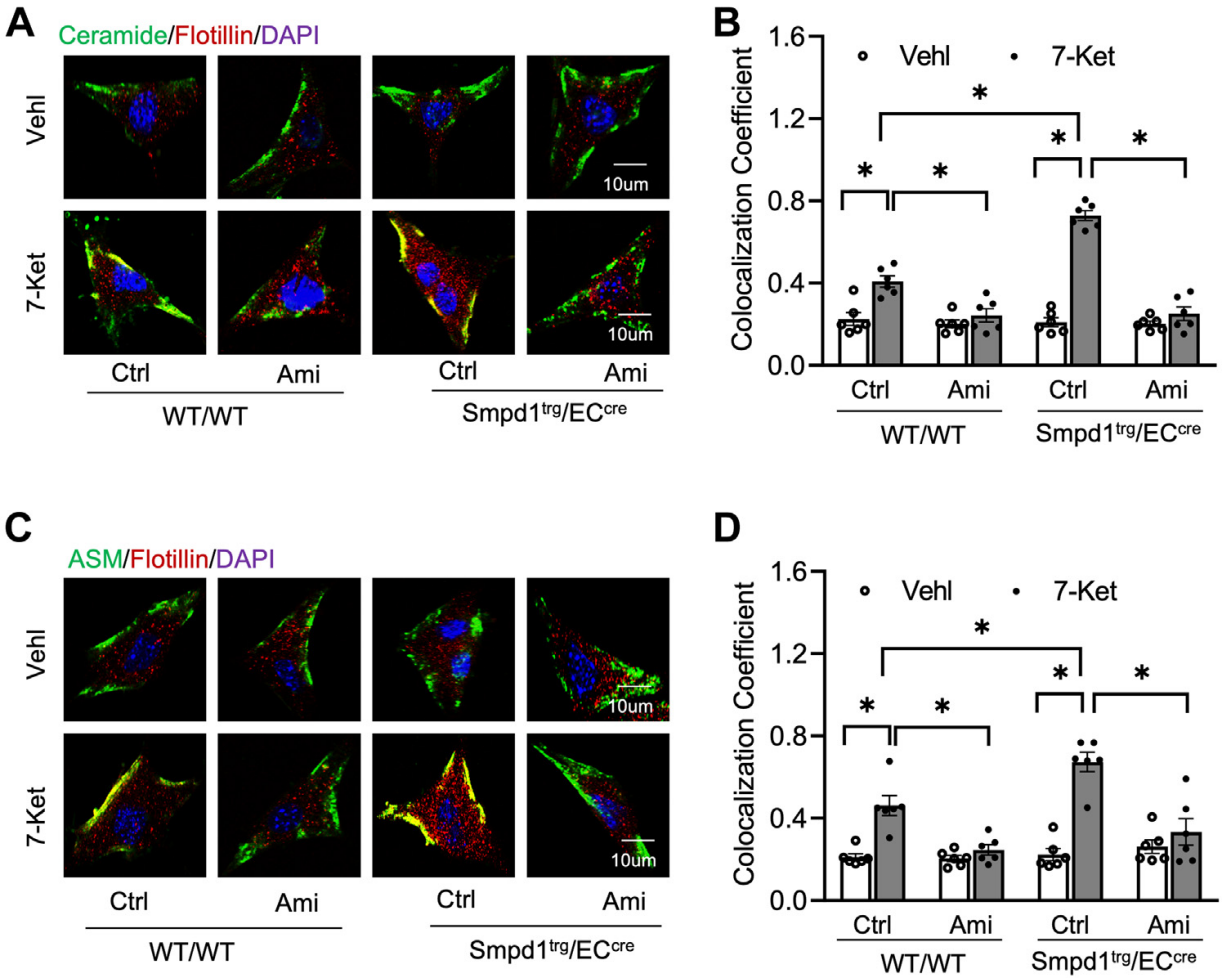
Formation of ASM-ceramide MR signaling platforms in the primary cultures of ECs from EC-specific *Smpd1* transgenic mice. A: Representative fluorescent confocal microscopic images showing the colocalization of MR marker, flotillin (red) with ASM (green). B: Summarized data showing the colocalization coefficient of flotillin with ASM. C: Representative fluorescent confocal microscopic images showing the colocalization of MR marker, flotillin (red) with ceramide (green). D: Summarized data showing the colocalization coefficient of flotillin with ceramide. Data are expressed as means ± SEM, *n* = 5. ∗*P* < 0.05 is defined as significant. The scale bar represents 10 μm.

As shown in Figure 8A–D, untreated *Smpd1*^trg^/EC^cre^ ECs had a similar level of colocalization of ceramide or ASM with flotillin compared with that of WT/WT ECs, suggesting that *Smpd1* gene overexpression does not trigger ceramide-enriched MR clustering under basal condition (vehicle groups). Interestingly, 7-Ket stimulated the colocalization of ceramide or ASM expression with flotillin in WT/WT ECs. Such 7-Ket-induced effects were enhanced in *Smpd1*^trg^/EC^cre^ ECs, which were significantly inhibited by treating the ECs with ASM inhibitor Ami. Therefore, our data indicate that *Smpd1* gene overexpression could enhance 7-Ket-induced ceramide-enriched MR clustering in ECs.

**Fig. 9:**
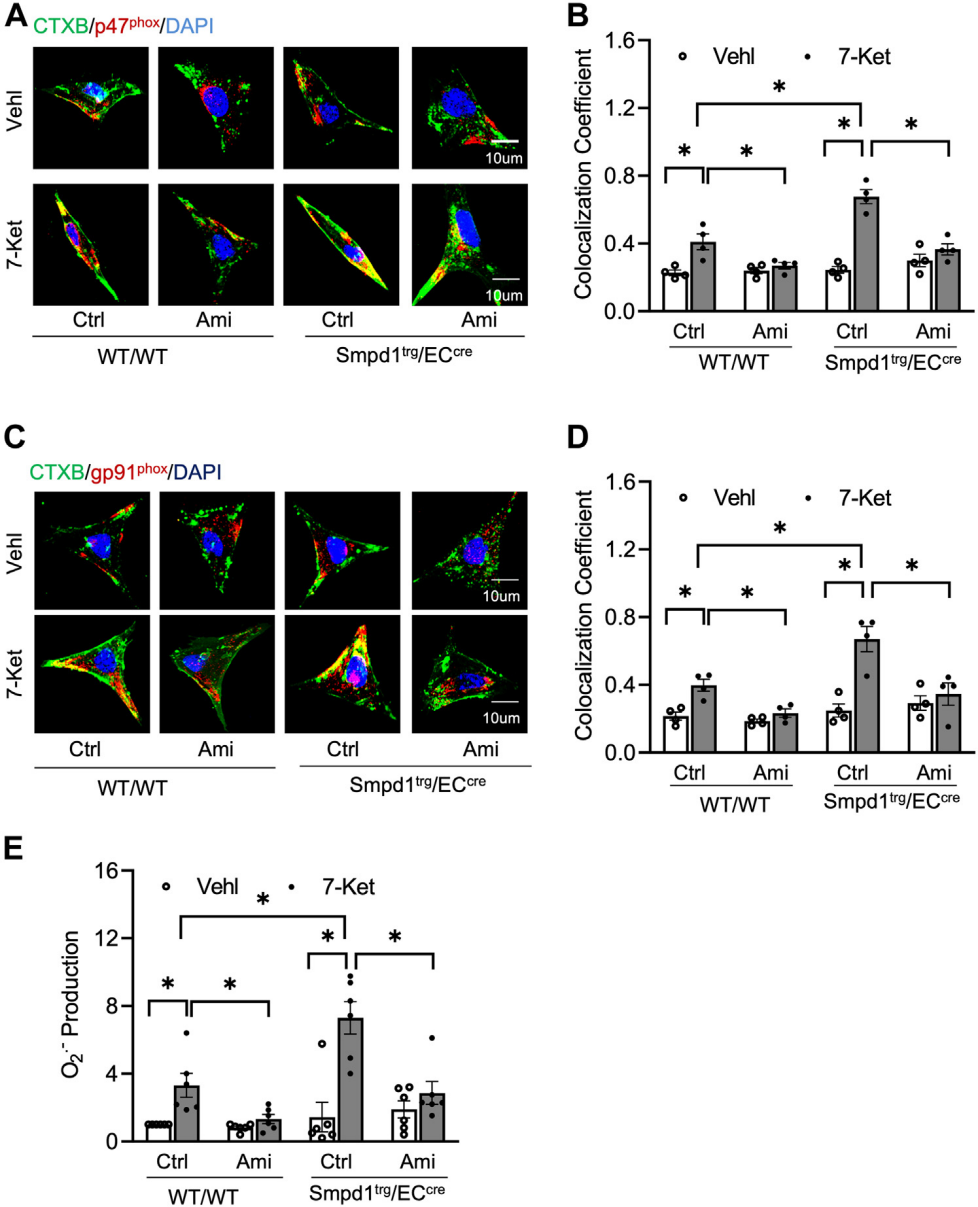
Aggregation and activation of NOXs in MR signaling platforms in the primary cultures of ECs from EC-specific *Smpd1* transgenic mice. A: Representative fluorescent confocal microscopic images showing the colocalization of MR marker, CTXB (green) with NOX subunit, gp47^phox^ (red). B: Summarized data showing the colocalization coefficient of CTXB with gp47^phox^. C: Representative fluorescent confocal microscopic images showing the colocalization of LR marker, CTXB (green) with NOX subunit, gp91^phox^ (red). D: Summarized data showing the colocalization coefficient of CTXB with gp91^phox^. E: NOX-mediated O_2_•^−^ production as measured by ESR spectrometry. Data are expressed as means ± SEM, *n* = 5. ∗*P* < 0.05 is defined as significant. The scale bar represents 10 μm.

We then examined the effects of *Smpd1* gene overexpression on the formation of MR-redox signaling platforms in ECs. MR was labeled with antibodies against CTXB (an MR marker). It was found that the expression of NOX subunits gp91 or gp47 in CTXB-labeled MR clusters was similar between WT/WT ECs and *Smpd1*^trg^/EC^cre^ ECs under ND conditions (control groups). 7-Ket increased the levels of colocalization of gp91^phox^ or gp47^phox^ with CTXB in WT/WT ECs, which were enhanced in *Smpd1*^trg^/EC^cre^ ECs and inhibited by Ami. Using ESR spectrometry, we measured the O_2_•^−^ production in cultured ECs. As shown in Figure 9E, 7-Ket increased O_2_•^−^ production in WT/WT ECs, an effect enhanced in *Smpd1*^trg^/EC^cre^ ECs. Treating both types of ECs with Ami significantly attenuated 7-Ket-induced O_2_•^−^ production. Together, these data suggest that *Smpd1* gene overexpression enhances the 7-Ket-induced formation of MR-redox signaling platforms in ECs.

### *Txnip* gene silencing abrogates 7-ket-increased NLRP3 inflammasome formation and activation in ECs with *Smpd1* gene overexpression

**Fig. 10:**
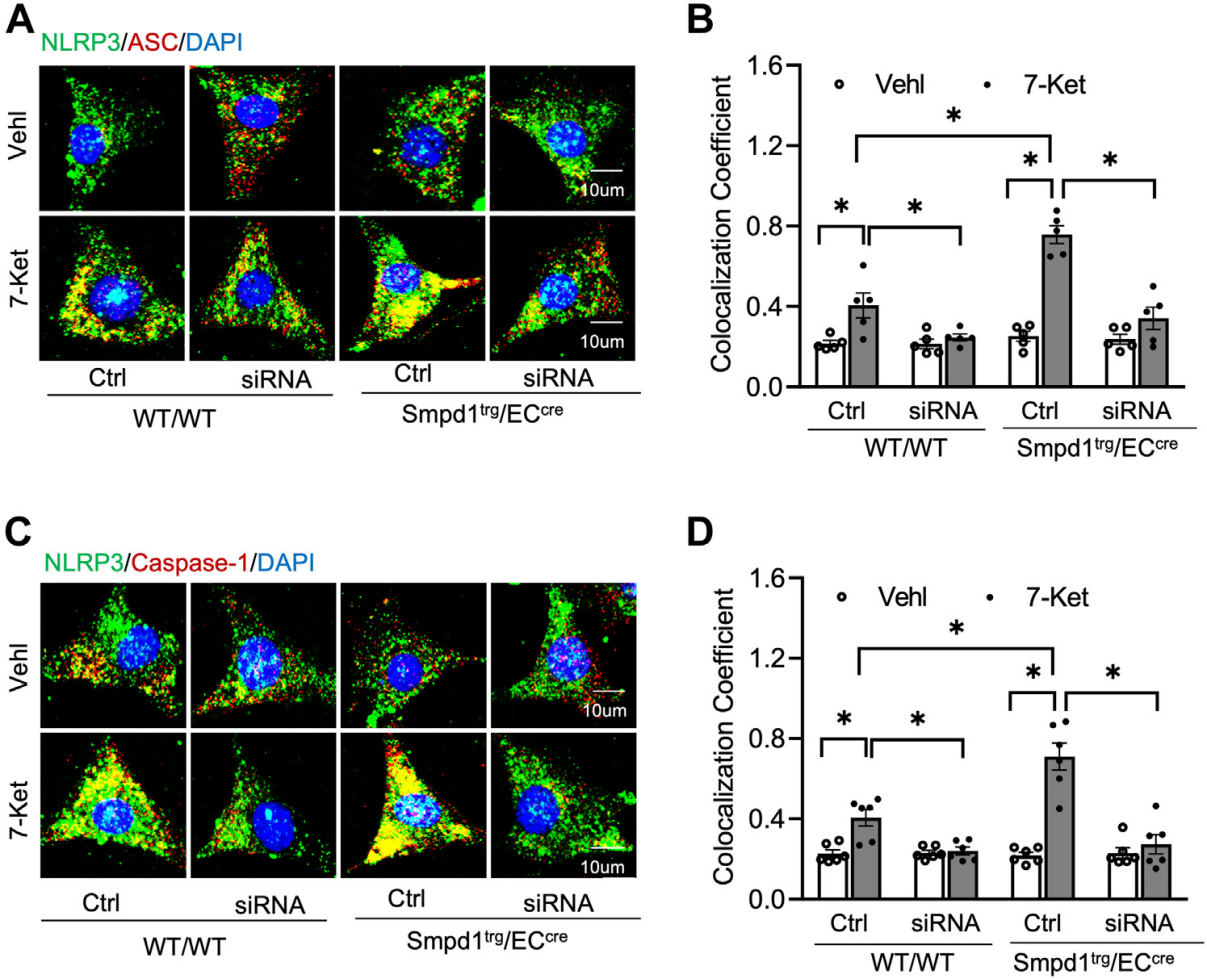
TXNIP gene silencing abrogated NLRP3 inflammasome formation in the primary cultures of ECs from EC-specific *Smpd1* transgenic mice. A: Representative fluorescent confocal microscope images displaying the yellow dots or patches showing the colocalization of NLRP3 (green) with ASC (red). B: The summarized data show the colocalization coefficient of NLRP3 with ASC. C: Representative fluorescent confocal microscope images showing the colocalization of NLRP3 (green) with caspase-1 (red). D: The summarized data show the colocalization coefficient of NLRP3 with caspase-1. Data are expressed as means ± SEM, *n* = 6. ∗*P* < 0.05 is defined as significant. The scale bar represents 10 μm.

**Fig. 11:**
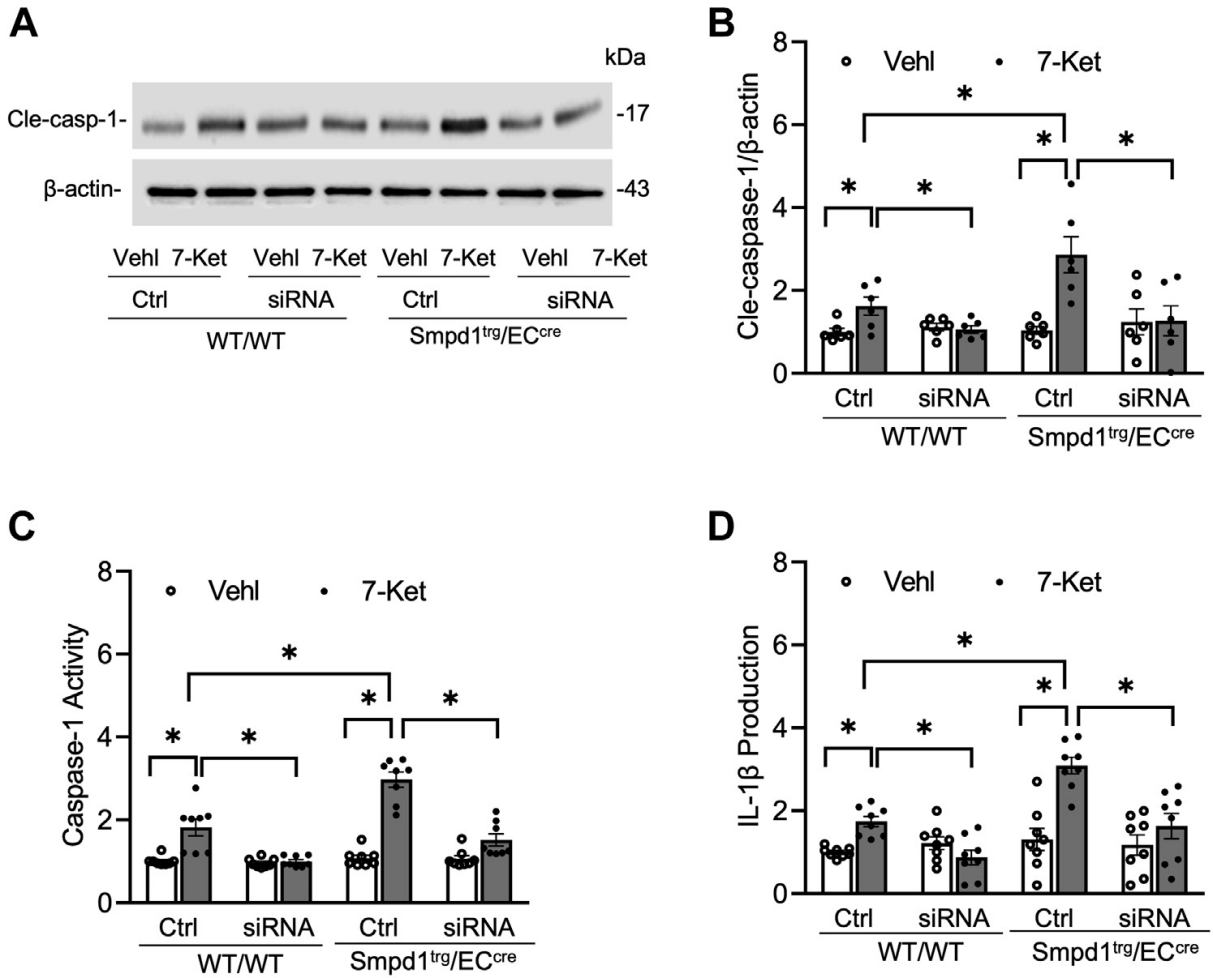
TXNIP gene silencing abrogated NLRP3 inflammasome activation in the primary cultures of ECs from EC-specific *Smpd1* transgenic mice. A: Representative Western blot gel documents showing the expression of cleaved caspase-1 in the primary cultures of carotid ECs from endothelium-specific *Smpd1* transgenic mice. B: Summarized data showing the expression of cleaved caspase-1 in the primary cultures of carotid ECs from EC-specific *Smpd1* transgenic mice. C: Summary data showing caspase-1 activity in the primary cultures of carotid ECs from EC-specific *Smpd1* transgenic mice. D: Summarized data showing IL-1β production in the primary cultures of carotid ECs from endothelium-specific *Smpd1* transgenic mice. Data are expressed as means ± SEM, *n* = 5. ∗*P* < 0.05 is defined as significant.

Previous studies demonstrated that TXNIP binds NLRP3 protein and triggers NLRP3 inflammasome formation and activation (43). Here, we examined the role of TXNIP in 7-Ket-induced NLRP3 inflammasome formation and activation in *Smpd1*^trg^/EC^cre^ ECs. As shown in Figure 10A–D, Txnip gene silencing by Txnip siRNA transfection markedly attenuated 7-Ket-induced increases in the colocalization of NLRP3 with ASC or caspase-1 in both WT/WT ECs and *Smpd1*^trg^/EC^cre^ ECs. 7-Ket-induced increases in caspase-1 cleavage, caspase-1 activity, and IL-1β production were significantly abolished by Txnip gene silencing in WT/WT ECs or *Smpd1*^trg^/EC^cre^ ECs (Fig. 11A–D). Together, these results demonstrate that targeting the *T*xnip gene abrogated 7-Ket-induced NLRP3 inflammasome activation and formation in ECs with *Smpd1* gene overexpression.

## Discussion

The present study revealed the critical contribution of endothelial ASM-ceramide MR redox signaling pathway to hypercholesterolemia-induced NLRP3 inflammasome activation and neointimal hyperplasia. Our results demonstrated that EC-specific overexpression of the *Smpd1* gene enhanced hypercholesterolemia-induced neointimal formation, which was accompanied by augmented ASM-ceramide-MR redox signaling and NLRP3 inflammasome formation and activation in the arterial endothelium. Moreover, we demonstrated that TXNIP is a critical mediator that links ASM-ceramide-MR redox signaling to NLRP3 inflammasome formation and activation in ECs. By using *Smpd1*^trg^/EC^cre^ mice and the primary cultures of their ECs, our findings provide the first direct evidence that ASM-ceramide-MR redox signaling and downstream redox sensor TXNIP are involved in the formation and activation of NLRP3 inflammasome activation and subsequent neointimal hyperplasia during hypercholesterolemia.

Accumulating evidence has revealed that ASM and ceramide are crucially involved in cardiovascular physiology and pathophysiology (44, 45). During atherosclerosis, lysosome ASM-dependent formation of ceramide-enriched lipid macrodomains in ECs contributes to FasL-induced impairment of the vasodilator response (46, 47) and muscarinic-1 receptor-mediated coronary artery constriction (48), which are both major aggravating factors leading to subsequent atherosclerosis. Recent studies have demonstrated that ASM-derived ceramide mediates NLRP3 inflammasome activation in various tissues including the heart and kidney (49, 50, 51), which suggests NLRP3 inflammasome as one of the effector pathways downstream of the ASM-ceramide pathway. Our recent study demonstrated that ASM deficiency inhibited the endothelial NLRP3 inflammasomes and neointimal lesions (24), which supports the view that ASM-ceramide signaling is involved in endothelial NLRP3 inflammasome activation. However, it remains unknown whether the ASM-ceramide signaling in ECs is directly involved in endothelial NLRP3 inflammasome activation and neointimal lesion formation and how ASM regulates NLRP3 inflammasome activation in response to high cholesterol stimulation in vitro and in hypercholesteremia in vivo. In the present study, we generated EC-specific *Smpd1* transgenic mice (*Smpd1*^trg^/EC^cre^), in which the *Smpd1* gene was specifically overexpressed in ECs. Their littermate controls (WT/WT or *Smpd1*^trg^/WT) were used for the comparison of the studies. Our data first confirmed that *Smpd1* gene overexpression in ECs significantly enhanced the WD-induced neointima formation in the carotid artery wall (Fig. 1). Then, confocal microscopic analysis revealed that EC-specific *Smpd1* gene overexpression markedly enhanced WD-induced increases in NLRP3 inflammasome formation and activation as characterized by increased colocalization of NLRP3 inflammasome components (Figs. 2 and 3). Consistently, we observed that in *Smpd1*^trg^/EC^cre^ mice, the enhanced WD-induced NLRP3 inflammasome activation and neointimal formation were accompanied by higher levels of ASM protein expression and ceramides in the endothelium (Fig. 4). Similarly, using isolated ECs from *Smpd1*^trg^/EC^cre^ mice and WT controls, we confirmed that *Smpd1* gene overexpression enhanced NLRP3 inflammasome activation (Figs. 6 and 7) and ASM-ceramide signaling (Fig. 8) in cultured ECs stimulated by 7-Ket. Taken together, these results suggest that endothelial ASM-ceramide signaling promotes endothelial NLRP3 inflammasome activation during hypercholesterolemic stimulations, which contributes to the development of neointima formation or atherogenic pathology. To our knowledge, this current study provides the first evidence that endothelium-specific overexpression of ASM exaggerates endothelial NLRP3 inflammasome formation and activation upon pathological stimuli such as hypercholesterolemia.

It is known that ROS generation is one of the first identified triggers of NLRP3 inflammasome activation (52, 53, 54). ROS may be derived from endoplasmic reticulum stress, damaged mitochondria, and NOX, which is an intermediate factor involved in multiple signaling pathways and can trigger the activation of NLRP3 inflammasome (55). Our previous studies have for the first time defined a mechanism mediating NOX activation in response to many different stimuli, termed MR redox signalosomes. These MR signalosomes use MRs as a platform to transduce and amplify the redox signaling and are centered on the enzymatic NOX subunit clustering and activating to produce O_2_•^−^ (56). NOX-derived ROS can act downstream to transduce transmembrane or intracellular signaling, leading to the redox regulation of cell and organ function (16). We reported that different stimuli such as homocysteine, visfatin, or ATP act on the cell membrane to stimulate ASM to produce and form ceramide-enriched MR platforms and thereby increase NOX-dependent O_2_•^−^ production in different types of cells, such as podocytes, ECs, and hepatic stellate cells (12, 17, 57, 58, 59, 60, 61, 62). So far, it remains poorly understood whether ASM-ceramide-MR redox signaling platforms are critical in the activation of NLRP3 inflammasomes and atherosclerotic lesions. Using ASM global knockout mice, we recently demonstrated that ASM/ceramide-associated MR clustering with NOX subunits contributes to hypercholesteremia-induced endothelial NLPR3 inflammasome activation and formation (24). In the present study, we further used *Smpd1*^trg^/EC^cre^ mice to study the specific role of ASM in the formation and activation of NLRP3 inflammasome and explore related molecular mechanisms. It was found that *Smpd1* gene overexpression enhanced WD-induced formation of ceramide-enriched MR redox signaling as shown by increased formation of flotillin-1-positive MR clusters aggregated with ASM/ceramide (Fig. 4) or NOX subunits gp91 and p47 (Fig. 5) and the consequent production of O_2_•^−^ levels as measured by in situ DHE staining. DHE is well known as a fluorescent probe for superoxide and hydrogen peroxide and is commonly used for the detection of ROS generation in tissues. Our data demonstrated that WD increased arterial fluorescence in the arterial wall including in ECs and SMCs of the media layer, which was further increased when the *Smpd1* gene is overexpressed. It is not surprising to observe DHE-ROS fluorescence in the media layer as hydrogen peroxide is cell permeable and could be released into the medial layer. The ROS production in the media layer could also be originated from redox pathways in SMCs that are activated following endothelial activation or injury. Nonetheless, our data indicate that EC-specific *Smpd1* gene overexpression enhanced the oxidative stress in the arterial wall. In addition, we confirmed that the *Smpd1* gene overexpression enhanced 7-Ket-induced activation of ASM-MR redox signaling in cultured ECs, which were abolished by the ASM inhibitor (Figs. 8 and 9). Together, these results from the ASM overexpression studies provide direct evidence that hypercholesterolemia or cholesterol stimulation instigates the ASM-ceramide pathway to induce MR redox signaling platform formation in ECs and thereby trigger endothelial NLRP3 inflammasomes.

To further study how ASM-mediated activation of MR redox signaling platforms induces NLRP3 inflammasome activation, we determine the role of a redox sensor TXNIP in the process of NLRP3 inflammasome activation. TXNIP is the endogenous inhibitor and regulator of thioredoxin, a major cellular antioxidant, and antiapoptotic system (63). It has been demonstrated that NLRP3 inflammasome activators induce the dissociation of TXNIP from thioredoxin in a ROS-sensitive manner, which allows thioredoxin to bind NLRP3, thereby activating this inflammasome in different cells, such as macrophages (64), ECs (65, 66, 67, 68), and podocytes (69, 70). However, the role of TXNIP in ASM-ceramide-mediated NLRP3 inflammasome activation remains largely unexplored. In the present study, we examined the effects of *T*xnip gene silencing on the NLRP3 inflammasomes in cultured ECs isolated from WT and *Smpd1*^trg^/EC^cre^ mice. Our results demonstrated that Txnip gene silencing abrogated 7-Ket-induced NLRP3 inflammasome formation and activation in ECs from both WT and *Smpd1*^trg^/EC^cre^ (Figs. 10 and 11). These data demonstrate that TXNIP is a critical downstream mediator of ASM-ceramide-MR redox signaling that triggers endothelial NLRP3 inflammasome formation and activation. Consistent with our finding, Jiang *et al.* (71) recently reported that lipopolysaccharide/ATP-induced increase of ASM activity and accumulation of ceramide is attributed to TXNIP/NLRP3 inflammasome activation in J774A.1 cell and THP-1 macrophages. Koka *et al.* (24) have reported that TXNIP inhibitor verapamil attenuated 7-Ket or cholesterol crystal-induced increases in caspase-1 activity in ECs. In addition, metformin and many other compounds were used to lower aortic TXNIP levels in vivo or endothelial levels in vitro to block NLRP3 inflammasome activation and protect from endothelial dysfunction and cardiovascular risk factors (72, 73, 74, 75, 76). Nrf2 and AMPK are also believed to control the regulation of the NLRP3 inflammasome by the TRX-TXNIP complex (77, 78, 79). Concerning the mechanism responsible for the action of TXNIP, there is evidence that it activates the TLR4-NFκB-NLRP3 inflammasome signaling pathway with increased MyD88, NLPR3 inflammasome, and ASC expression, as well as the increased phosphorylation of IκBα and p65, thus promoting downstream NF-κB activation (80). The overexpression of TXNIP also led to an increased expression of inflammation genes via chromatin modifications and by promoting nuclear translocation of NF-κB (81). These previous studies have indicated that TXNIP may perform its functions through multiple binding partners and thereby directly or indirectly regulate NLRP3 inflammasome formation and activation. The precise mechanism by which TXNIP links ASM-ceramide-MR redox signaling and its downstream effector, NLRP3 inflammasome, deserves future investigation.

Finally, the present study had several limitations, which should be noted. First, our lipid panel analysis demonstrated that *Smpd1*^trg^/EC^cre^ mice had increased lipid levels induced by the WD. The mechanism by which serum lipid is increased by endothelial overexpression of ASM remains an intriguing question. Recent evidence identifies that liver sinusoidal EC dysfunction is the main characteristic or early event in the pathogenesis of the nonalcohol fatty liver disease, which contributes to impaired hepatic lipid uptake and metabolism (82). Thus, endothelial ASM overexpression in the liver may cause liver sinusoidal EC dysfunction and hepatic dysregulation of lipid metabolism leading to higher serum lipids, which indirectly contribute to the development of neointimal injury. Second, the EC-specific Smpd1 transgene (*Smpd1*^trg^/EC^cre^) mice were generated by crossing *Smpd1*^trg^/WT mice with EC-specific Tie2-Cre mice as described (40). Tie2 is a receptor tyrosine kinase that binds angiopoietin-1 and angiopoietin-2. Tie2 is not only expressed in ECs (83) but also found in hematopoietic cells in fetal liver and adult bone marrow and several differentiated hematopoietic cells (84, 85). The formation and activation of NLRP3 inflammasome in hematopoietic cells are implicated in cardiovascular diseases (86, 87). In this regard, some of the effects may be mediated by ASM overexpression in the hematopoietic cells on cardiovascular diseases.

In summary, the present study demonstrated that EC-specific *Smpd1* gene overexpression enhanced the production of ceramide and the formation of MR redox signaling platforms, promoted the activation of endothelial NLRP3 inflammasomes, and thereby resulted in endothelial dysfunction and atherogenesis. These findings provide the first evidence that endothelial ASM-ceramide-MR redox signaling is directly linked with endothelial NLRP3 inflammasomes and neointimal hyperplasia. Moreover, we identified that ASM-ceramide MR redox signaling is coupled with endothelial NLRP3 inflammasome activation via a redox sensor, TXNIP. Our findings may offer novel therapeutic insights into targeting ASM-ceramide-MR redox signaling or TXNIP to suppress the activation of endothelial NLRP3 inflammasomes, thereby preventing and treating vasculopathy associated with hypercholesterolemia.

## Data Availability

The data that support the findings of this study are available from the corresponding author on realistic request.

## Supplemental Data

This article contains supplemental data.

## Conflict of Interest

The authors declare that they have no conflicts of interest with the contents of this article.

## Figures and Tables

**Figure S3:**